# Random effects modelling *versus* logistic regression for the inclusion of cluster-level covariates in propensity score estimation: A Monte Carlo simulation and registry cohort analysis

**DOI:** 10.3389/fphar.2023.988605

**Published:** 2023-03-23

**Authors:** Mike Du, Albert Prats-Uribe, Sara Khalid, Daniel Prieto-Alhambra, Victoria Y. Strauss

**Affiliations:** ^1^ Botnar Research Centre, Nuffield Orthopaedic Centre, Nuffield Department of Orthopaedics, Rheumatology, and Musculoskeletal Sciences, University of Oxford, Oxford, United Kingdom; ^2^ Boehringer-Ingelheim Pharma GmbH & Co., KG, Ingelheim, Germany

**Keywords:** propensity score, simulation, trial emulation, clustered data, random effects model, causal inference

## Abstract

**Purpose:** Surgeon and hospital-related features, such as volume, can be associated with treatment choices and outcomes. Accounting for these covariates with propensity score (PS) analysis can be challenging due to the clustered nature of the data. We studied six different PS estimation strategies for clustered data using random effects modelling (REM) compared with logistic regression.

**Methods:** Monte Carlo simulations were used to generate variable cluster-level confounding intensity [odds ratio (OR) = 1.01–2.5] and cluster size (20–1,000 patients per cluster). The following PS estimation strategies were compared: i) logistic regression omitting cluster-level confounders; ii) logistic regression including cluster-level confounders; iii) the same as ii) but including cross-level interactions; iv), v), and vi), similar to i), ii), and iii), respectively, but using REM instead of logistic regression. The same strategies were tested in a trial emulation of partial *versus* total knee replacement (TKR) surgery, where observational *versus* trial-based estimates were compared as a proxy for bias. Performance metrics included bias and mean square error (MSE).

**Results:** In most simulated scenarios, logistic regression, including cluster-level confounders, led to the lowest bias and MSE, for example, with 50 clusters × 200 individuals and confounding intensity OR = 1.5, a relative bias of 10%, and MSE of 0.003 for (i) compared to 32% and 0.010 for (iv). The results from the trial emulation also gave similar trends.

**Conclusion:** Logistic regression, including patient and surgeon-/hospital-level confounders, appears to be the preferred strategy for PS estimation.

## Introduction

Observational studies using routinely collected patient data from health registries are often used for clinical treatment comparative studies when randomised control trials are unfeasible or unethical (Bernard et al., 2014). Conversely to randomisation in trials, treatment allocation in observational data is often driven by patient and physician features, leading to confounding by indication. First proposed by Rosenbaum and Rubin, propensity score (PS) weighting is a popular method to minimise the resulting bias (Rosenbaum and Rubin, 1983; Rosenbaum and Rubin, 1984). Most PS applications in pharmacoepidemiology include only patient covariates. Conversely, medical devices and surgical studies typically have a clustered structure that accommodates hospital and physician/surgeon features that could impact treatment and outcome and hence act as confounders (Montgomery and Schneller, 2007; Papachristofi et al., 2017).

Several simulation studies on PS weighting have shown that using random effects models (DerSimonian and Kacker, 2007) in the PS estimation or treatment outcome modelling can reduce the bias arising from cluster-level confounding in clustered data (Arpino and Mealli, 2011; Li et al., 2013; Schuler et al., 2016; Yang, 2018; Cafri and Austin, 2020; Fuentes et al., 2021). However, it is unclear what is the best strategy for PS estimation when the outcome is estimated using the random effect model in observational studies of medical devices or surgical procedures. Therefore, this study aims to evaluate different PS estimation strategies for weighting, given the random effects model is used to estimate the treatment outcome.

We used Monte Carlo simulations (Raychaudhuri, 2008) and a surgical trial emulation study comparing partial and total knee replacement (TKR) surgery to evaluate the accuracy and precision of REM compared to logistic regression for PS estimation. Additionally, we tested the impact of such PS estimation strategies in different scenarios of cluster-level confounding intensity (weak to strong) and cluster sizes.

## Methods

### Simulation data generation process

**FIGURE 1:**
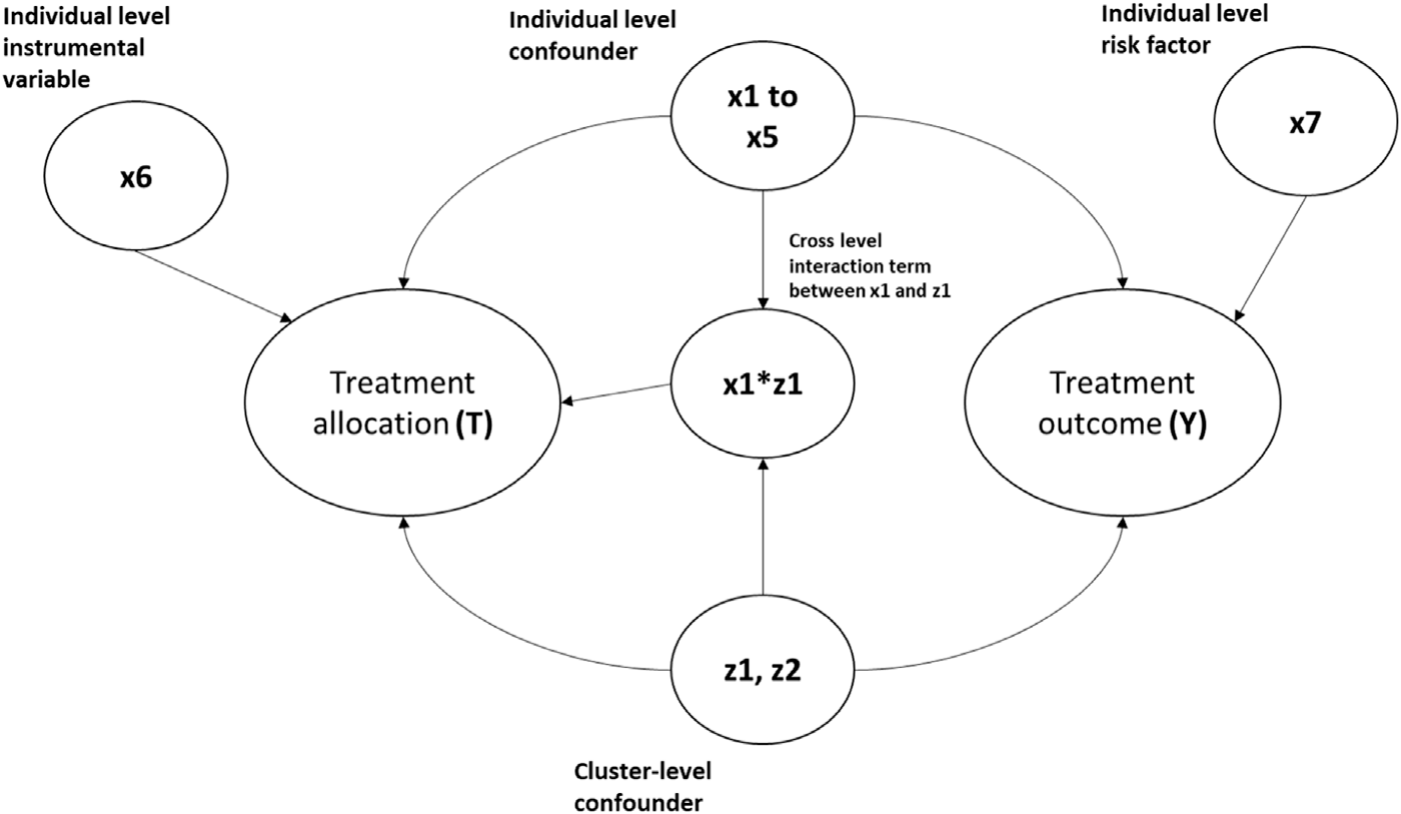
Diagram showing the causal relationship between the covariates in the simulation data. The arrows indicate causes. For example, x1-> Y implies x1 causes Y.

The simulation settings were based on previous simulation studies (Arpino and Mealli, 2011; Li et al., 2013) but with parameters adapted to medical device/surgical epidemiology data. We simulated clustered datasets *via* Monte Carlo simulations with an average sample size of 10,000 individuals to represent the patients, binary treatment allocation (T), and binary outcome (Y). We simulated six patient-level covariates (x1–x6), two cluster-level covariates (z1 and z2 to represent potential hospital- and surgeon-level confounders), and a cross-level interaction term between the individual and cluster-level confounders for each patient. Among the individual covariates simulated, five were confounders (x1–x5), one (x6) was an instrumental variable associated with the exposure but not with the outcome, and x7 was a risk factor associated with the outcome but not with exposure. Both cluster-level covariates (z1 and z2) were generated as confounders. Treatment (T) and outcome (Y) binary variables were then generated using a random intercept model with the simulated covariates. The complete mathematical formulae for data generation are included in the supplementary material. Figure 1 gives the clustered causal diagram of the simulation covariates.

Thirty-five different scenarios with 1,000 replications each were simulated to test: 1) five different combinations of cluster (*m*): average cluster size (*n*) 10: 1,000, 50: 200, 100:100, 200:50, and 500:20, where (*m*) represents the number of clusters and the number of individuals in the cluster was generated with a Poisson distribution with mean (*n*); 2) different effect size for z1 and z2 on the outcome, ranging from negligible with odds ratio = 1.01 (resembling an instrumental variable) to strong with odds ratio = 2.5 (equivalent to strong multilevel confounding); and 3) different effect size for the across-level interaction term on treatment allocation, ranging from negligible with odds ratio = 1.01 to strong with odds ratio = 2.5. Table 1 gives the generation distribution, effect on treatment allocation, and effect on treatment outcome for all the covariates generated in the simulations.

**TABLE 1:** Generation distribution, effects on treatment allocation, and effects on treatment outcomes for covariates generated in the simulations. OR, odds ratio.

Covariate	Description	Effects on treatment allocation (beta value)	Effects on treatment outcome (beta value)	Generation distribution
z1, z2	Cluster-level confounders	z1 = z2 = 0.4055 (equivalent to OR = 1.5)	z1 = z2 = [0.01, 0.2231, 0.4055, 0.9163]	z1 ∼ N (0, 1)
			∼ [equivalent to OR = 1.01, 1.25, 1.5, 2.5]	z2 ∼ Bernoulli (0.5)
x1–x5	Individual-level confounders	[x1, x2, x3, x4, x5] = [0.35, 0.4, 0.45, 0.5, 0.55]	[x1, x2, x3, x4, x5] = [0.35, 0.4, 0.45, 0.5, 0.55]	[x1, x2, x3]∼Bernoulli [(0.4, 0.45, 0.5)] x4, x5 ∼ N (0,1)
x6	Individual-level risk factor	0	0.5	Bernoulli (0.5)
x7	Individual-level instrumental variable	0.5	0	Bernoulli (0.5)
z1*x1	Cross-level interaction term	[0.01, 0.2231, 0.4055, 0.9163] ∼ [equivalent to OR = 1.01, 1.25, 1.5, 2.5]	0	z1*x1

Furthermore, we have included 15 additional scenarios where the data’s cluster size (*n*) was generated *via* a negative binomial distribution with dispersion parameters of 0.1 and 0.2 rather than the Poisson distribution because the range of cluster sizes generated with the Poisson distribution can be limited. Using the negative binomial distribution, we can generate clusters with a larger range of cluster sizes, which helps us determine the robustness of the simulation results and strengthen its generalisability.

The simulation data were generated using the simstudy (version 0.2.1) R package, and the PS models were fitted using the lme4 (version 1.1.21) R package.

### Propensity score estimation strategy

For all the data scenarios described in the simulation data generation process, we tested six different strategies to estimate PS, as defined in Table 2 (M1–M6).

**TABLE 2:** Cluster-level information contained and the statistical models used for the six propensity score estimation strategies (M1–M6).

Propensity score strategy	Cluster-level confounders as covariates in the PS model	Cross-level confounder interaction term as a covariate in the PS model	Statistical model to build a propensity score
M1	Excluded	Excluded	Logistic regression
M2	Included	Excluded	Logistic regression
M3	Included	Included	Logistic regression
M4	Excluded	Excluded	Random effects model^a^
M5	Included	Excluded	Random effects model^a^
M6	Included	Included	Random effects model^a^

^a^ The random effects models were built with a logit link function.

### Treatment effect estimation

For each of the scenarios, the average treatment effect (ATE) was estimated using random effects models with a logit function regressed on treatment outcome weighted with stabilised inverse probability weighting (SIPW) (Xu et al., 2010) based on PS calculated using each of the strategies described in Table 2. Random effects models were used for treatment effect estimation as several simulation studies on PS (Arpino and Mealli, 2011; Li et al., 2013) have shown that the use of random effects models to account for the cluster-level confounding generally gives the least bias.

### Assessment of simulation results

We measured each PS specification strategy’s performance on each scenario by calculating 1) relative bias (%), defined as the average percentage difference between the true treatment effect and the estimated treatment effect; 2) mean square error (MSE), as a measure of accuracy; and 3) confidence interval coverage, defined as the proportion of the 95% confidence intervals of the estimated treatment effects containing the true treatment effect. All the performance measures were calculated following the simulation study guidelines discussed by Morris et al. (2019) using the “rsimsum” (version 0.9.1) R package.

We decided not to name reference PS strategies M1–M6 in this paper because there is no clear guidance in the current literature. We could say that M2 would be the preferred method in general as it included all the known confounders from the sample in the PS model, which is the general method for non-clustered data. However, we could also argue that the cluster-level confounders might behave differently from patient-level confounders in PS estimation, and the cluster-level confounding has already been dealt with in the outcome estimation stage with the random effects outcome model. These arguments would also make the use of M1 completely valid.

### Case study on medical device and surgical epidemiology

We used data from the UTMOST study (Prats-Uribe et al., 2021), which aimed to identify optimal methods for controlling confounding when emulating the results of the TOPKAT surgical trial (Beard et al., 2019). The UTMOST cohort study included patients with a first primary TKR or unicompartmental knee replacement (UKR) (Wilson et al., 2019) in the UK National Joint Registry (NJR) from 2009 to 2016 who met the TOPKAT trial eligibility criteria. UTMOST included 2,94,556 patients (2,94,556 TKR and 21,026 UKR) and 6,420 lead surgeons carrying out the interventions. UTMOST extracted patient-level covariates from the UK NJR, linked to Hospital Episode Statistics (HES) records and to the NHS PROMS (patient-reported outcome measures) database; surgeon volume of UKR performed by each lead surgeon in the previous year was obtained from the NJR. The UTMOST study outcome was revised 5 years after surgery. Table 3 details the covariates included in the study.

**TABLE 3:** Covariates adjusted in the case study.

Covariate	Type/description
Socio-demographic covariates	Patient covariates (individual-level)
Age	Continuous covariate
Gender	Binary covariate
Rural, urban	Categorical covariate—urban/town and fringe/village/isolated
IMD	Categorical covariate in 10 percentiles from least deprived to most deprived
BMI	Continuous covariate
Pre-operative patient reported outcomes	Patient covariates (individual-level)
Pre-operative OKS	Continuous covariate
EQ-5D^a^	Continuous
General health	Categorical covariate with discrete scale excellent/1/2/3/4/poor
Comorbidities 3 years before surgery	Patient covariates (individual-level)
Charlson comorbidity	Binary covariate
Gastrointestinal disease	Binary covariate
Osteoarthritis and other joint problems	Binary covariate
Mental health	Binary covariate
Respiratory disease	Binary covariate
Cardiovascular disease	Binary covariate
Thyroid problems	Binary covariate
Foot, hip, and spinal pain	
Foot, hip, and spinal pain	Binary covariate
Coxarthrosis	Binary covariate
Neurological disorders	Binary covariate
Other arthroses	Binary covariate
Polyarthrosis	Binary covariate
Spondylosis	Binary covariate
Surgeon’s feature covariates	Surgeon covariates (cluster-level)
Surgery volume of UKR performed by each lead surgeon in the previous year from the NJR	Continuous covariate

UKR, unicompartmental knee replacement; NJR, national joint registry.

^a^ Standardised measure of health-related quality of life developed by the EuroQol Group.

We applied the six proposed PS specification strategies from Table 2 to the UTMOST dataset to construct PS for UKR and compared the findings to those of the TOPKAT surgical trial. The cross-level interaction term considered in UTMOST was the interaction of surgeon volume and patient gender. As with the simulated data described in the Methods section, we modelled 5-year revision risk for patients receiving UKR using a random effects model with the lead surgeon as cluster-level and patient-level covariates using SIPW.

## Results

### Simulation study

**FIGURE 2:**
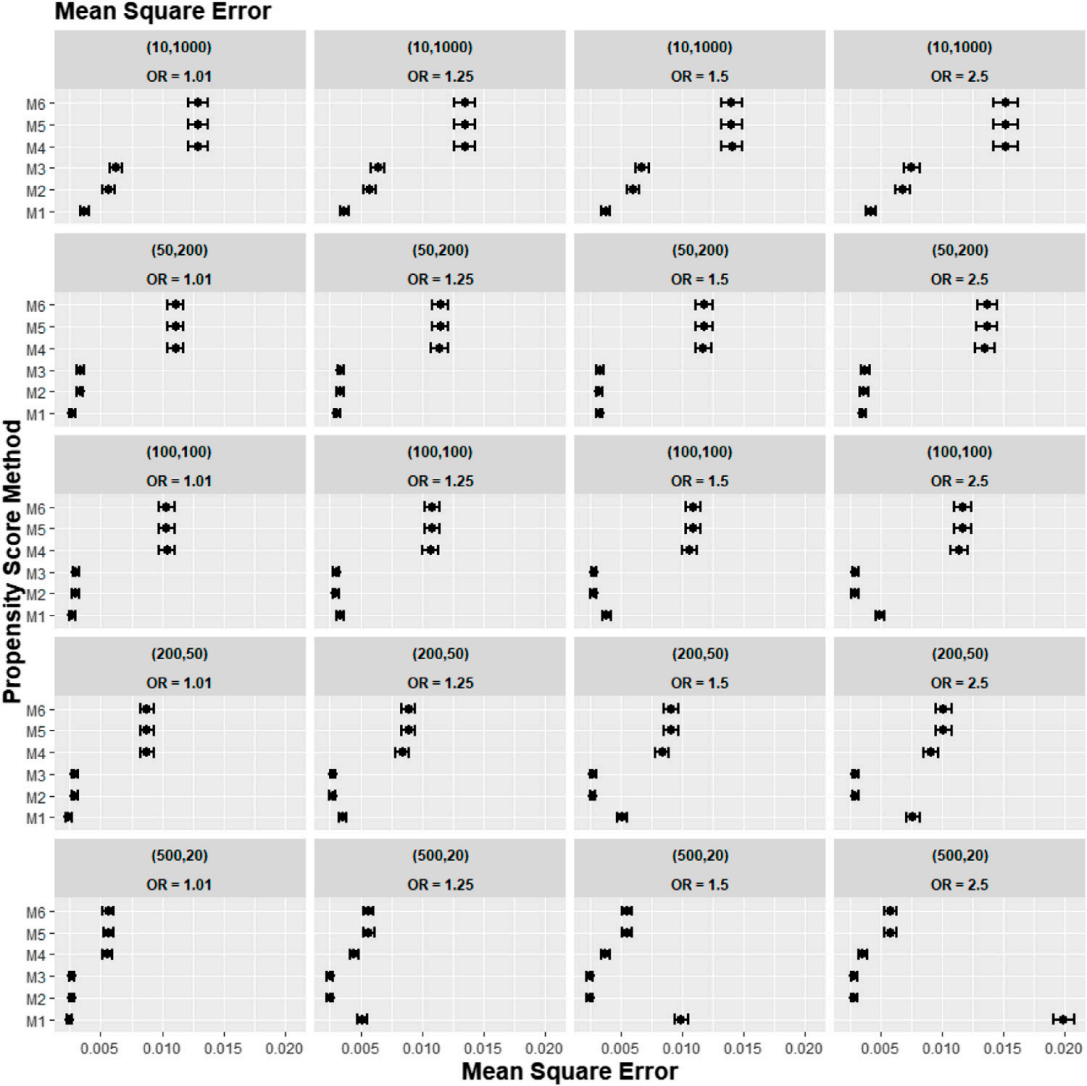
Graphs showing the simulation treatment effects’ average MSE and 95% confidence interval for propensity score specification strategies M1–M6 for different cluster-structure and cluster- (surgeon-) level confounder odds ratios on treatment outcomes. Structure = (number of clusters, individuals per cluster) and surgeon OR = cluster-level confounder odds ratio on treatment outcomes. Propensity score (PS) strategies: M1, logistic regression PS excluding cluster-level confounders; M2, logistic regression PS including cluster-level confounders; M3, logistic regression PS with cluster-level confounders and the cross-level interaction term; M4, random effects PS excluding cluster-level confounders; M5, random effects PS including cluster-level confounders; M6, random effects PS with cluster-level confounders and the cross-level interaction term.

**FIGURE 3:**
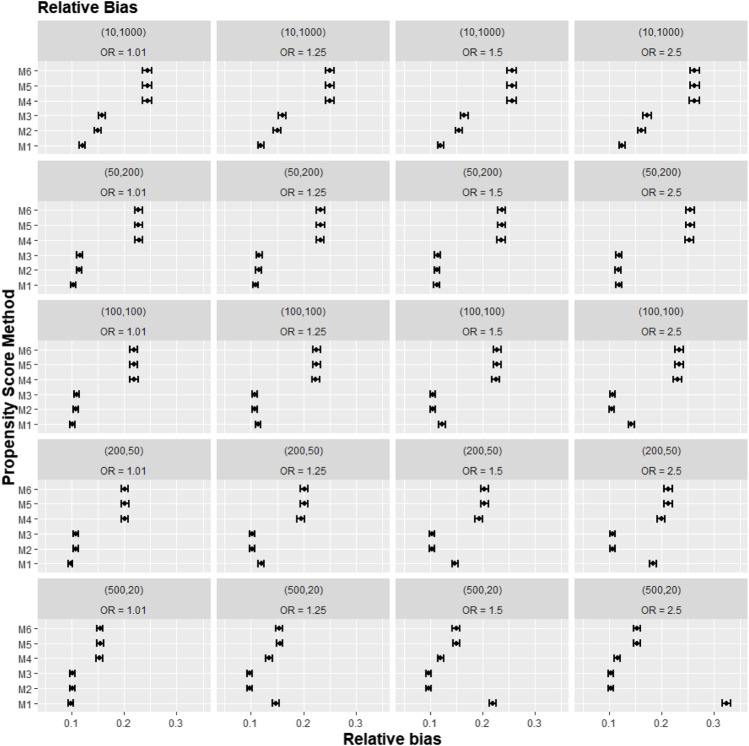
Graphs showing the simulation treatment effects’ average absolute relative bias and 95% confidence interval for propensity score specification strategies M1–M6 for different cluster-structure and cluster- (surgeon-) level confounder odds ratios on treatment outcomes. Structure = (number of clusters, individuals per cluster) and surgeon OR = cluster-level confounder odds ratio on treatment outcomes. Propensity score (PS) strategies: M1, logistic regression PS excluding cluster-level confounders; M2, logistic regression PS including cluster-level confounders; M3, logistic regression PS with cluster-level confounders and the cross-level interaction term; M4, random effects PS excluding cluster-level confounders; M5, random effects PS including cluster-level confounders; M6, random effects PS with cluster-level confounders and the cross-level interaction term.

**FIGURE 4:**
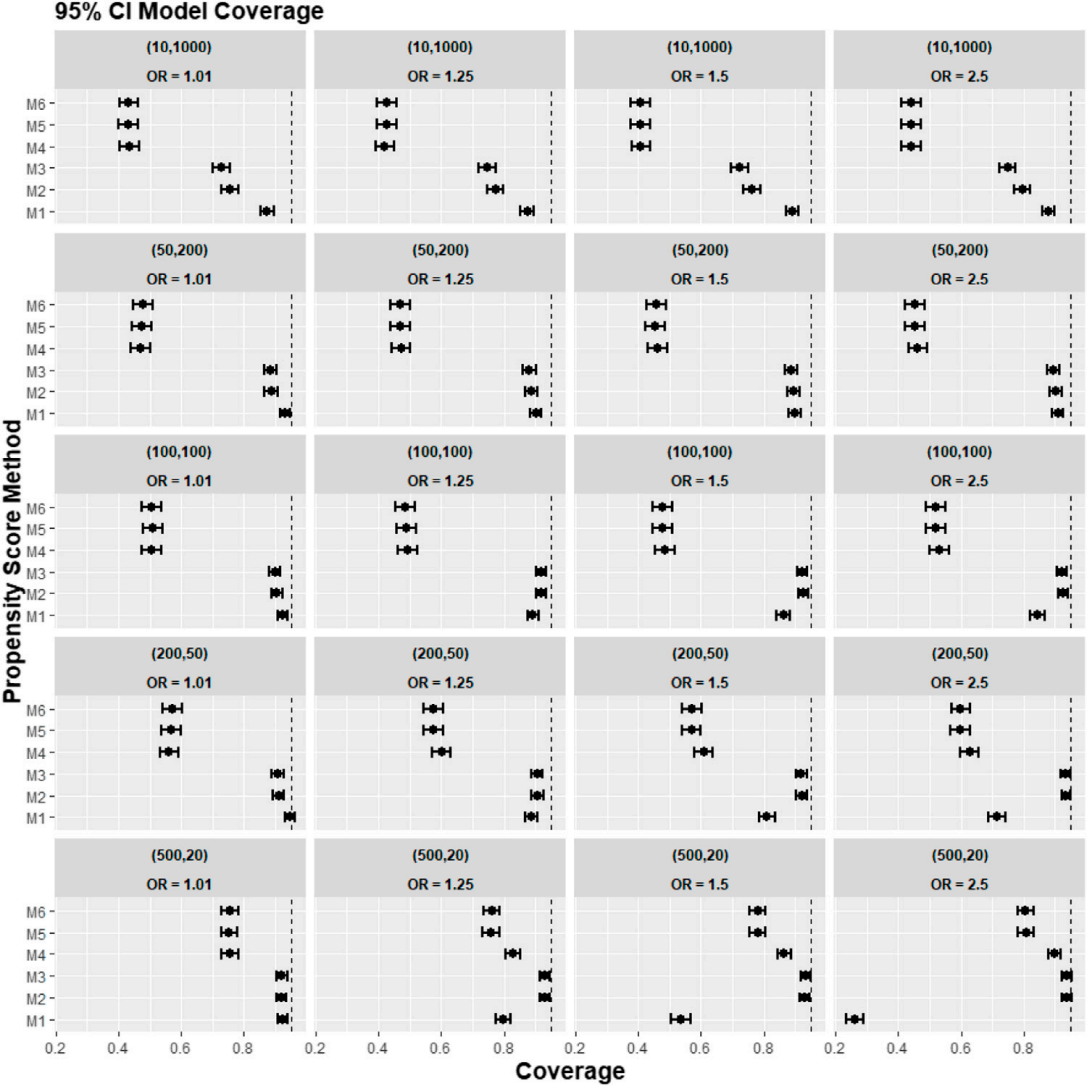
Graphs showing the simulation treatment effects’ average 95% CI model coverage probability and its 95% confidence interval for propensity score specification strategies M1–M6 for different cluster-structure and cluster-level confounder odds ratios on treatment outcomes. Structure = (number of clusters, individuals per cluster) and surgeon OR = cluster-level confounder odds ratio on treatment outcomes. Propensity score (PS) strategies: M1, logistic regression PS excluding cluster-level confounders; M2, logistic regression PS including cluster-level confounders; M3, logistic regression PS with cluster-level confounders and the cross-level interaction term; M4, random effects PS excluding cluster-level confounders; M5, random effects PS including cluster-level confounders; M6, random effects PS with cluster-level confounders and the cross-level interaction term. The black vertical dotted line indicates 95%.

Figures 2–4 gave the simulations’ average absolute relative bias and MSE of the treatment effect estimates for PS estimation strategy M1–M6. Few clear trends were consistent in all cluster-structure scenarios, as shown in Figures 2, 4. The relative bias and MSE for models with and without the cross-level interaction were similar; for example, relative bias = 10.3% in M2 and relative bias = 10.4% in M3 for cluster-level confounders’ odds ratio (OR) = 1.01 and cluster-structure (m = 100, n = 100) scenario, suggesting not incorporating the cross-level correlation where one did not significantly impact bias. In scenarios where cluster-level confounders had minimal effect on outcome (OR = 1.01), the model omitting cluster-level confounders in the logistic-based PS (M1) gave the lowest bias. In contrast, M1 led to more bias as cluster-level confounding became stronger (OR ≥ 1.5) and where cluster size was smaller (Figure 3).

Apart from the result from the cluster structure (*m* = 500, *n* = 20), the random effects modelling- (REM)-based PS (M4, M5, and M6) gave consistently higher bias compared to logistic-based PS (M1, M2, and M3) in all other cluster–structure scenarios. For example, the relative bias for M4 is 24.5% compared to 11.4% for M1 in cluster-structure (*m* = 50, *n* = 200) and cluster-level confounder effect size odds ratio 1.5 scenario. Furthermore, adding the cluster-level confounders as covariates in the PS model did not impact the bias much in cluster number (*m*) and cluster size (*n*) = [ (10, 1000), (50, 200)] scenarios, regardless of the cluster-level confounder effect size on the treatment outcome. As Figure 2 showed that the relative bias observed in M1 was similar to that of M2 and M3, while the relative bias observed in M4 was similar to that of M5 and M6.

The results for the smallest two cluster-size scenarios (*m* = 500, *n* = 20) and (*m* = 200, *n* = 50) behaved differently compared to the other cluster structures tested in the study. Apart from the cluster confounder effect on outcome OR = 1.01 scenario, where the relative bias for M1 were smallest. The relative bias for the PS strategy that included the cluster-level confounders as covariates in the PS model (M2–M3) reduced bias compared to the PS strategy that did not consider the cluster level (M1). The improvement in bias and MSE was greater as the cluster-level confounder effect on outcome increased and was also greater as the cluster sizes of the data decreased. For example, the relative bias for M1 = 11.4% compared to M2 = 9.77% for the cluster-level confounder effect on outcome OR = 1.25 in the cluster-structure (*m* = 200, *n* = 50) scenario. For cluster-level confounder effect size on outcome OR = 2.5 in the cluster structure (*m* = 500, *n* = 20), the relative bias for M1 = 31.7% compared to M2 = 9.86%.

Figure 4 reports the coverage for the simulation study. Our experiments showed that coverage was lower for treatment effect estimates using REM-based PS (M4–M6), particularly in large cluster-size scenarios (*n* ≥ 50). In our small cluster-size scenario (*n* = 20), coverage in M4 was more similar to that seen using strategies M1, M2, and M3. However, M2 and M3 still gave higher model coverage than M4.

**FIGURE 5:**
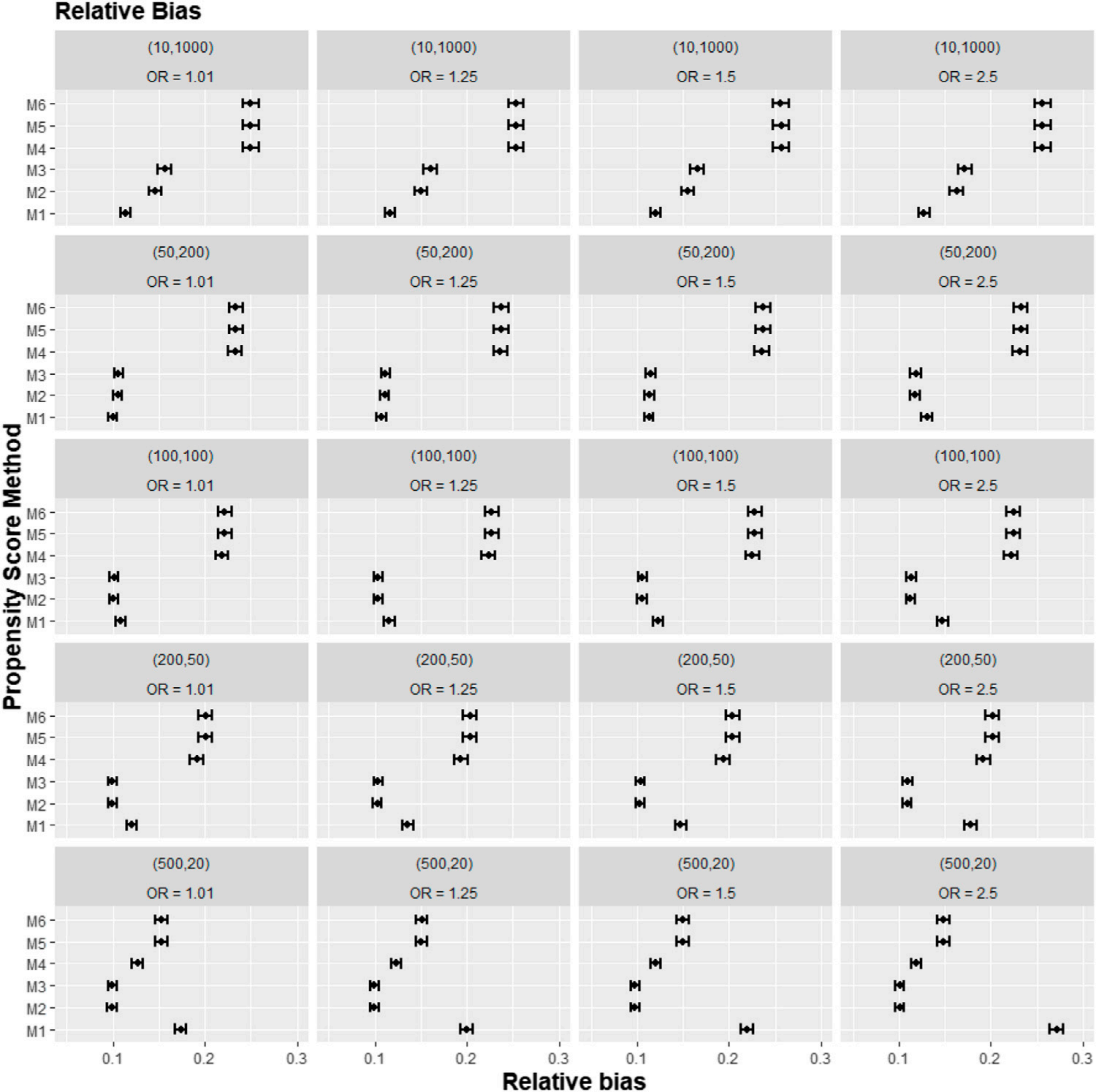
Graphs showing the simulation treatment effects’ average absolute relative bias and 95% confidence interval for propensity score specification strategies M1–M6 for different cluster-structure and cross-level interaction effects in the odds ratio on treatment allocation. Structure = (number of clusters, individuals per cluster) and surgeon OR = cluster-level confounder odds ratio on treatment outcomes. Propensity score (PS) strategies: M1, logistic regression PS excluding cluster-level confounders; M2, logistic regression PS including cluster-level confounders; M3, logistic regression PS with cluster-level confounders and the cross-level interaction term; M4, random effects PS excluding cluster-level confounders; M5, random effects PS including cluster-level confounders; M6, random effects PS with cluster-level confounders and the cross-level interaction term.

**FIGURE 6:**
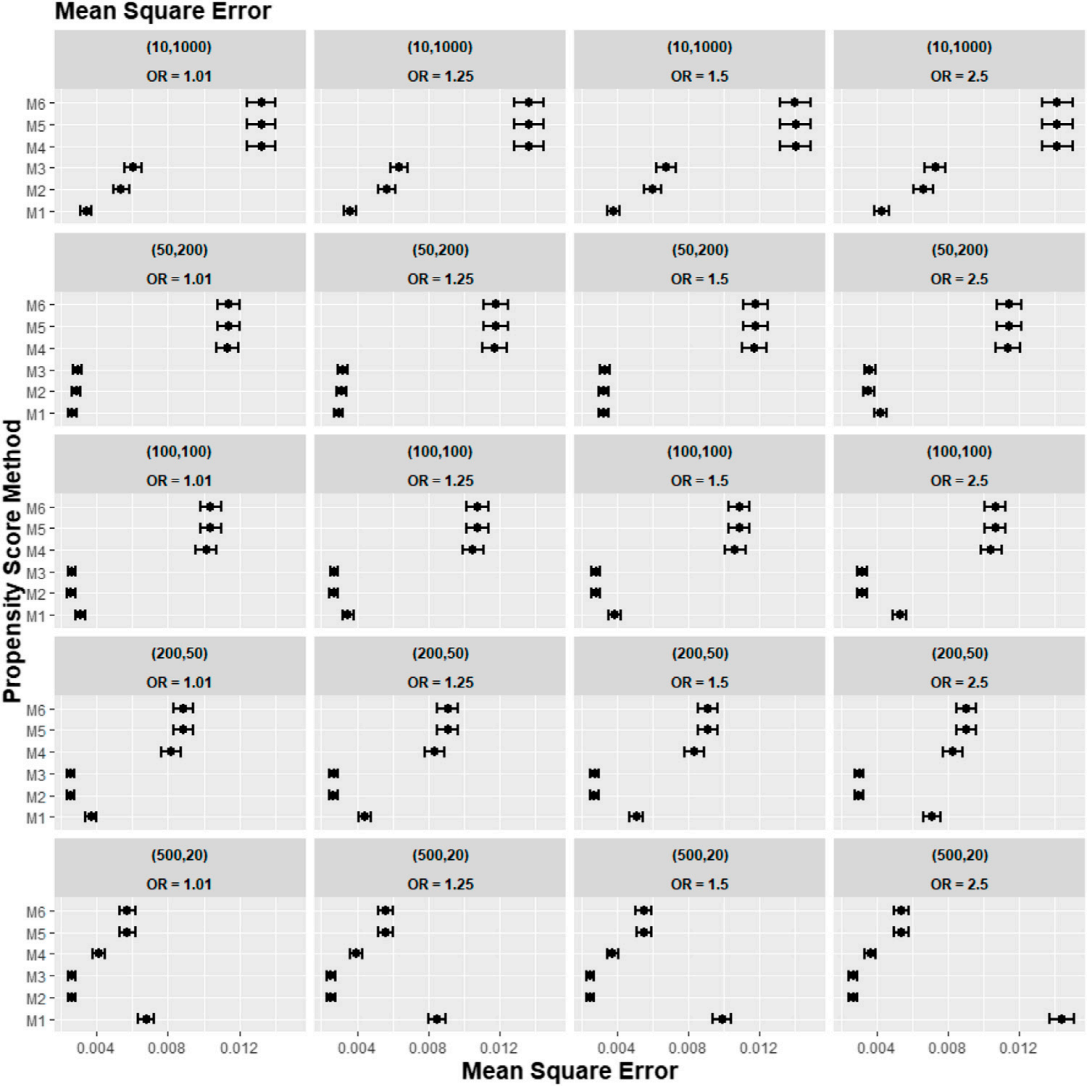
Graphs showing the simulation treatment effects’ average MSE and 95% confidence interval for propensity score specification strategies M1–M6 for different cluster-structure and cross-level interaction effects in the odds ratio on treatment allocation. Structure = (number of clusters, individuals per cluster) and surgeon OR = cluster-level confounder odds ratio on treatment outcomes. Propensity score (PS) strategies: M1, logistic regression PS excluding cluster-level confounders; M2, logistic regression PS including cluster-level confounders; M3, logistic regression PS with cluster-level confounders and the cross-level interaction term; M4, random effects PS excluding cluster-level confounders; M5, random effects PS including cluster-level confounders; M6, random effects PS with cluster-level confounders and the cross-level interaction term.

**FIGURE 7:**
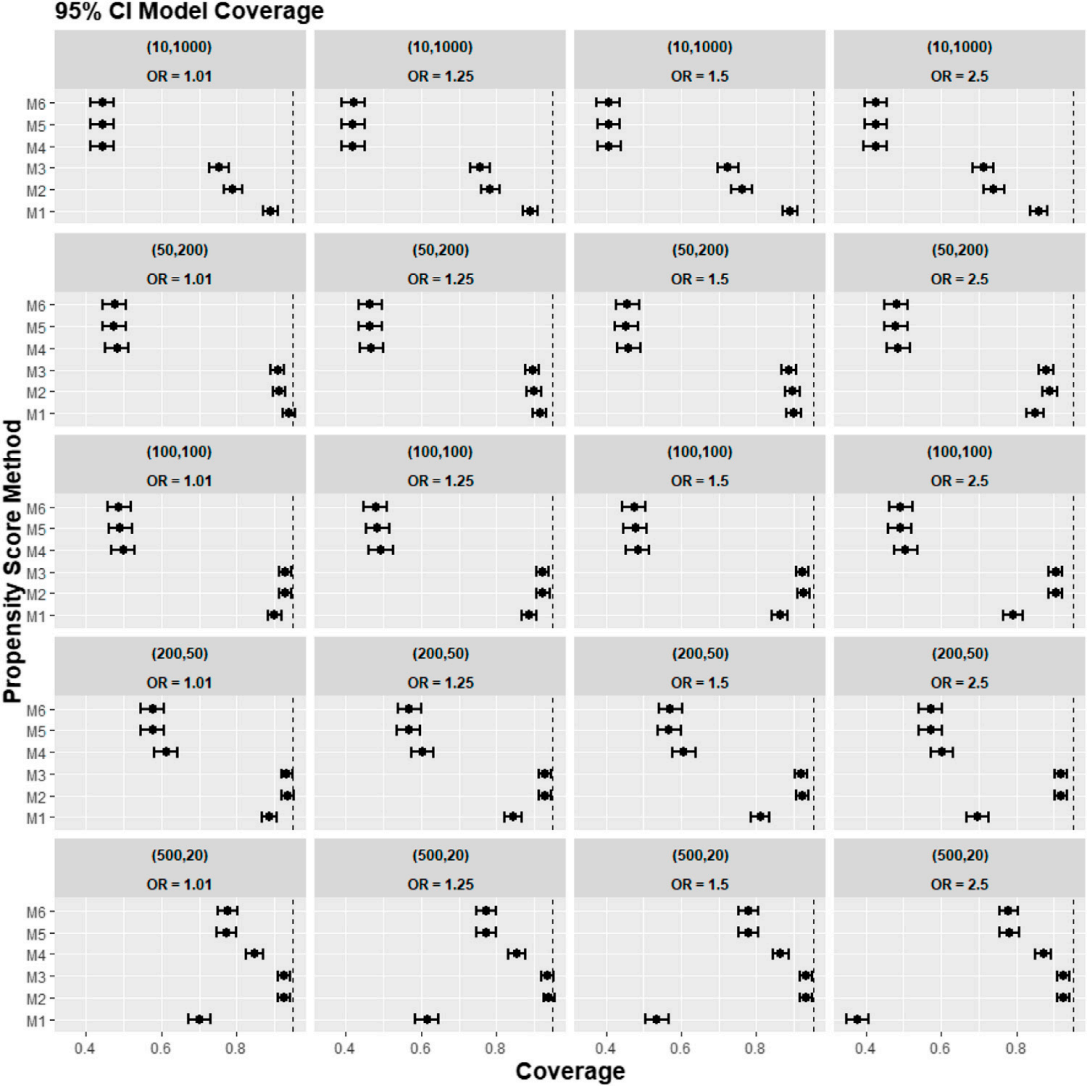
Graphs showing the simulation treatment effects’ average 95% CI model coverage probability and its 95% confidence interval for propensity score specification strategies M1–M6 for different cluster-structure and cross-level interaction effects in the odds ratio on treatment allocation. Structure = (number of clusters, individuals per cluster), and surgeon OR = cluster-level confounder odds ratio on treatment outcomes. Propensity score (PS) strategies: M1, logistic regression PS excluding cluster-level confounders; M2, logistic regression PS including cluster-level confounders; M3, logistic regression PS with cluster-level confounders and the cross-level interaction term; M4, random effects PS excluding cluster-level confounders; M5, random effects PS including cluster-level confounders; M6, random effects PS with cluster-level confounders and the cross-level interaction term. The black vertical dotted line indicates 95%.

A further set of simulation results is shown in Figures 5–7, where we varied the cross-level interaction effect on treatment while keeping the cluster-level confounder effect on treatment outcome at OR = 1.5. The results in relative bias, MSE, and 95% CI coverage were similar to those in Figures 2–4. Hence, the results suggested that regardless of the strength of the cross-level interaction term effect size on treatment allocation, the cross-level interaction term in the PS model made little impact on the treatment effect estimates.

**FIGURE 8:**
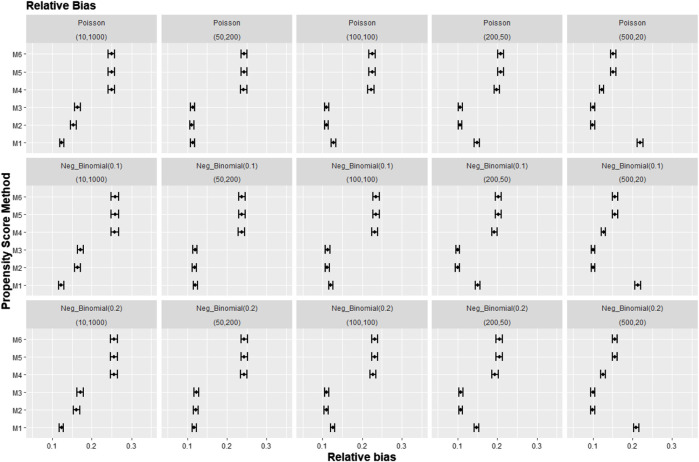
Graphs showing the simulation treatment effects’ average absolute relative bias and 95% confidence interval for propensity score specification strategies M1–M6 for different cluster structures and cluster sizes generated with different probability distributions for cluster-level confounder effect on outcome OR = 1.5. The mean of the probability distribution is the same as the average number of individuals per cluster. Poisson, Poisson distribution; Neg_binomial(0.1), negative binomial distribution with dispersion parameter 0.1; Neg_binomial (0.2), negative binomial distribution with dispersion parameter 0.2; Structure = (number of clusters, individuals per cluster). Propensity score (PS) strategies: M1, logistic regression PS excluding cluster-level confounders; M2, logistic regression PS including cluster-level confounders; M3, logistic regression PS with cluster-level confounders and the cross-level interaction term; M4, random effects PS excluding cluster-level confounders; M5, random effects PS including cluster-level confounders; M6, random effects PS with cluster-level confounders and the cross-level interaction term.

**FIGURE 9:**
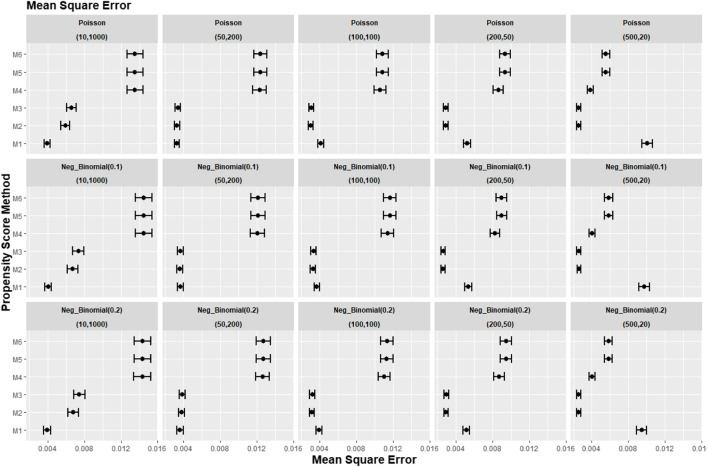
Graphs showing the simulation treatment effects’ average MSE and 95% confidence interval for propensity score specification strategies M1–M6 for different cluster structures and cluster sizes generated with different probability distributions for cluster-level confounder effect on outcome OR = 1.5. The mean of the probability distribution is the same as the average number of individuals per cluster. Poisson, Poisson distribution; Neg_binomial (0.1), negative binomial distribution with dispersion parameter 0.1; Neg_binomial (0.2), negative binomial distribution with dispersion parameter 0.2. Structure = (number of clusters, individuals per cluster). Propensity score (PS) strategies: M1, logistic regression PS excluding cluster-level confounders; M2, logistic regression PS including cluster-level confounders; M3, logistic regression PS with cluster-level confounders and the cross-level interaction term; M4, random effects PS excluding cluster-level confounders; M5, random effects PS including cluster-level confounders; M6, random effects PS with cluster-level confounders and the cross-level interaction term.

**FIGURE 10:**
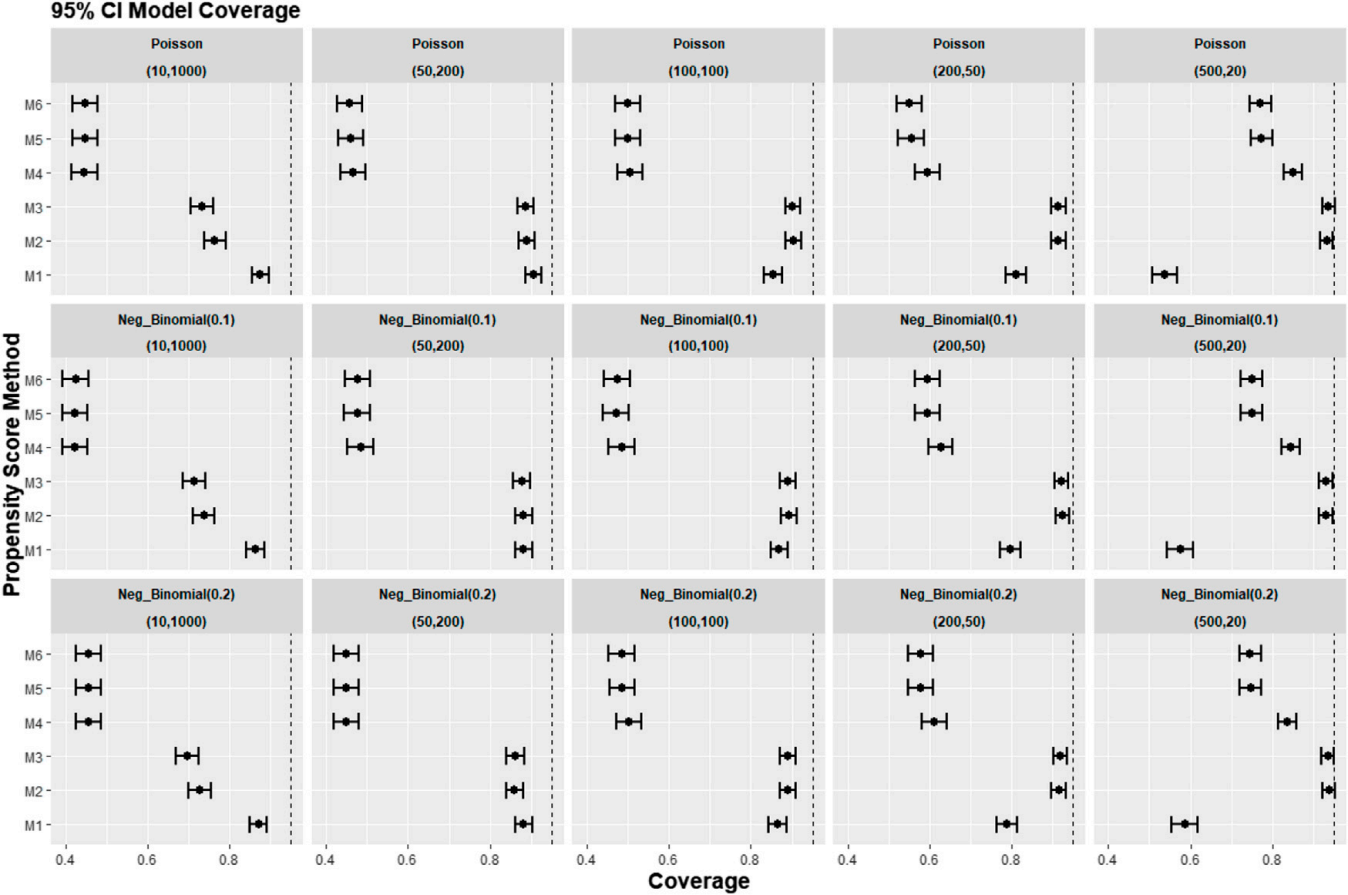
Graphs showing the simulation treatment effects’ average 95% CI model coverage probability and 95% confidence interval for propensity score specification strategies M1–M6 for different cluster structures and cluster sizes generated with different probability distributions for cluster-level confounder effect on outcome OR = 1.5. The mean of the probability distribution is the same as the average number of individuals per cluster. Poisson, Poisson distribution; Neg_binomial (0.1), negative binomial distribution with dispersion parameter 0.1; Neg_binomial (0.2), negative binomial distribution with dispersion parameter 0.2. Structure = (number of clusters, individuals per cluster). Propensity score (PS) strategies: M1, logistic regression PS excluding cluster-level confounders; M2, logistic regression PS including cluster-level confounders; M3, logistic regression PS with cluster-level confounders and the cross-level interaction term; M4, random effects PS excluding cluster-level confounders; M5, random effects PS including cluster-level confounders; M6, random effects PS with cluster-level confounders and the cross-level interaction term.

Figures 8–10 show the results for scenarios where we varied the cluster size range in the simulated data with negative binomial distribution for cluster-level confounder effect on treatment outcome and allocation at OR = 1.5. The results in relative bias, MSE, and 95% CI coverage were similar regardless of the distribution used, suggesting that our simulation results are likely to be robust against the change in the variability of the cluster sizes.

### Real-world case study

**FIGURE 11:**
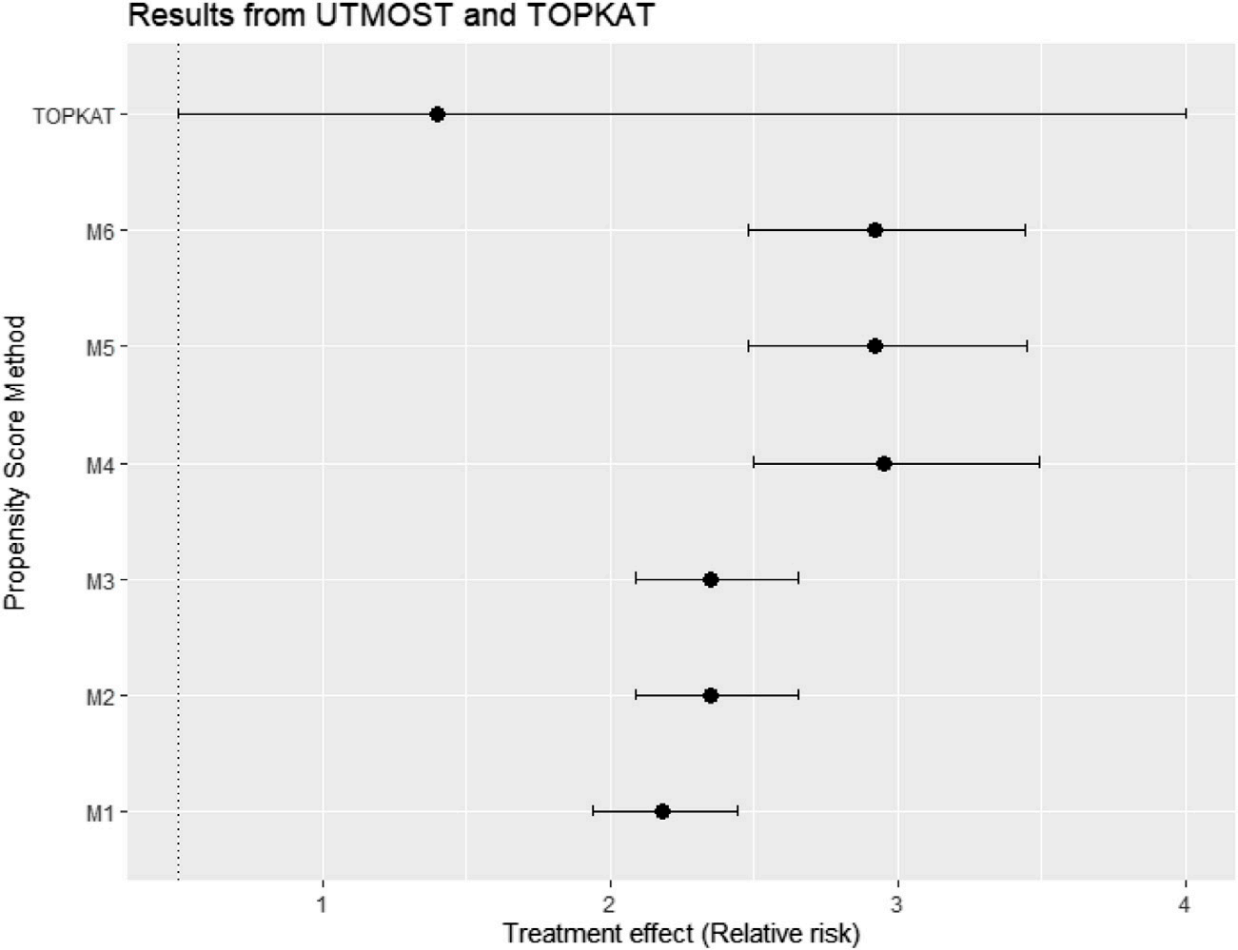
Treatment effect estimates in relative risk and their 95% confidence interval using data from the UTMOST study and the six proposed propensity score strategies M1–M6 and also the TOPKAT surgical trial estimates. TOPKAT, surgical trial estimates. Propensity score (PS) strategies: M1, logistic regression PS excluding cluster-level confounders; M2, logistic regression PS including cluster-level confounders; M3, logistic regression PS with cluster-level confounders and the cross-level interaction term; M4, random effects PS excluding cluster-level confounders; M5, random effects PS including cluster-level confounders; M6, random effects PS with cluster-level confounders and the cross-level interaction term.

Figure 11 gives the treatment effect estimates using the six PS strategies (M1–M6) proposed for the case study (UTMOST) and the TOPKAT surgical trial estimates. We found that, under all model strategies, UKR had a higher risk for 5-year revision than TKR. In contrast, TOPKAT found no statistically significant difference in the revision risk between UKR and TKR. Models that incorporated multilevel data or not and/or included the cluster-level confounders in the PS model had an overlapping confidence interval of outcome estimates. This meant all six proposed PS strategies (M1–M6) gave similar treatment estimates and were not statistically significantly different. In addition, PS models with and without the cross-level interaction term had similar estimates (M2 *vs*. M3, M5 *vs*. M6), suggesting that adding the cross-level interaction term in the PS models did not impact the estimate.

## Conclusion and Discussion

### Discussion

This study aimed to find the best way to account for cluster-level confounding in the PS model for PS weighting analysis when the random effects model was used to estimate the treatment outcome. In the simulation study, we found accounting for the cluster-level confounders in the PS model when the random effects model was used as the outcome model does not always give the smallest bias. For cluster structures with small cluster numbers and large cluster sizes (*m* = 10, *n* = 1,000) and (*m* = 50, *n* = 200), a strategy that ignored the cluster-level confounders (M1) performed the best. The inclusion of the cluster-level confounders in the PS model using the random effects model and as covariates in the model only offered noticeable improvement in bias for small cluster-size scenarios [e.g., (*m* = 500, *n* = 20) and (*m* = 200, *n* = 50)]. We could hypothesise that when the cluster size is small, cluster-level covariates act more similarly to patient-level covariates (e.g., the cluster-level covariates would be the same as patient-level covariates in data with one patient per cluster). Hence, failure to include cluster-level confounders would more likely cause bias in the study with a small cluster size. This is also consistent with previous studies on PS for clustered data (Arpino and Mealli, 2011; Li et al., 2013; Fuentes et al., 2021), which showed that the random effects model might give more accurate estimation in PS compared to the logistics regression model but not improvement in accuracy for treatment effect estimation. In addition, we found that adding the cross-level interaction term made little impact on the treatment effect in the simulation study. Thus, our simulation study showed that the optimal PS model strategy depended on the clustered structure and cluster-level confounder effect on the outcome. However, previous simulation studies (Li et al., 2013; Fuentes et al., 2021) on this topic were more focused on the performance of different weighting approaches.

Applying the proposed PS strategies to real-world clinical studies corroborated with some but not all of our simulation results. The inclusion of a cross-level interaction term in the logistic regression or random effects model did not substantially change the estimated treatment effect, the same as the simulation study result. However, the treatment effect estimates in the real-world clinical study all had overlapping confidence intervals, meaning all six PS strategies (M1–M6) are not significantly different, which differs from our simulation results. There were some differences between the cluster structure, which could contribute to these differences in the result. First, the cluster size distribution followed a Poisson distribution in the simulation study, but the distribution for the real-world clinical study did not. Second, we found that many surgeons carried out only one type of treatment in the real-world clinical study. However, in our simulation study, the treatment is allocated individually, meaning both treatments can appear in all clusters. This discrepancy in results between our real-world clinical study and simulations also highlighted that the cluster structure of the data affected the accuracy and precision of results for PS weighting analysis. More research is needed on how different cluster structures affect PS weighting analysis.

### Strengths and limitations to the study

This study’s main strength is its use of both simulation and real-world data. The use of simulated data, where the true average treatment effect was known, allowed us to compare the accuracy of the six proposed PS estimation strategies. The use of clinical data allowed us to test whether the trends from the simulation study were held with real-world data.

This study has several limitations. In the simulation study, we investigated only five different cluster-number and cluster-size scenarios. Hence, the results may not be generalisable to other cluster-number and cluster-size scenarios. In addition, we only tested the PS strategies on binary outcomes. Therefore, our results cannot generalise to other types of outcomes. We also assumed that the treatment assignment was influenced only by a small set of covariates in the simulation study. It could be argued that, in real-world settings, the data would usually contain more covariates. However, the focus of this study was not on the number of covariates. Finally, the TOPKAT trial treatment estimate was underpowered in the real-world case study. As a result, the 95% confidence interval for the trial treatment estimate was large, making it difficult to compare the accuracy of the treatment effects from the UTMOST data.

### Conclusion

In summary, careful consideration of the cluster structure is necessary to decide on the best model for PS estimation. We should only consider the use of the random effects model in the PS model when the dataset contains large numbers of small clusters. Moreover, we should consider including cluster-level confounders as covariates in the PS model, especially when the cluster-level confounders are thought to strongly affect the treatment outcome and there are a larger number of clusters in the data as this can reduce bias.

## Data Availability

The original contributions presented in the study are included in the article/supplementary material. Further inquiries can be directed to the corresponding author.
